# An e–Mental Health Resource for COVID-19–Associated Stress Reduction: Mixed Methods Study of Reach, Usability, and User Perceptions

**DOI:** 10.2196/39885

**Published:** 2022-08-26

**Authors:** Nadia Minian, Allison Gayapersad, Anika Saiva, Rosa Dragonetti, Sean A Kidd, Gillian Strudwick, Peter Selby

**Affiliations:** 1 Nicotine Dependence Service Centre for Addiction and Mental Health Toronto, ON Canada; 2 Department of Family and Community Medicine University of Toronto Toronto, ON Canada; 3 Institute of Medical Science University of Toronto Toronto, ON Canada; 4 Department of Pharmacology and Toxicology University of Toronto Toronto, ON Canada; 5 Centre for Addiction and Mental Health Toronto, ON Canada; 6 Department of Psychiatry University of Toronto Toronto, ON Canada; 7 Institute of Health Policy, Management and Evaluation University of Toronto Toronto, ON Canada; 8 Campbell Family Mental Health Research Institute Centre for Addiction and Mental Health Toronto, ON Canada; 9 Dalla Lana School of Public Health University of Toronto Toronto, ON Canada

**Keywords:** COVID-19, website, stress, mental health, eHealth, internet-based intervention, mixed methods evaluation, usability, digital health, health informatics

## Abstract

**Background:**

COVID-19 and its public health response are having a profound effect on people’s mental health. To provide support during these times, Canada’s largest mental health and addiction teaching hospital (Centre for Addiction and Mental Health [CAMH]) launched the Mental Health and COVID-19 Pandemic website on March 18, 2020. This website was designed to be a nonstigmatizing psychoeducational resource for people experiencing mild to moderate distress due to COVID-19 and the public health response to the pandemic.

**Objective:**

The aim of this study was to examine the reach, usability, and user perceptions of the CAMH Mental Health and COVID-19 Pandemic website.

**Methods:**

This study used a mixed methods sequential explanatory design approach, which consisted of the following 2 distinct phases: (1) quantitative data collection and analysis and (2) qualitative semistructured interviews. In phase 1, we analyzed Google Analytics data to understand how many people visited the website and which were the most visited pages. We conducted a survey to identify users’ sociodemographic backgrounds, and assess the usability of the website using the System Usability Scale and users’ subjective stress levels using the Perceived Stress Scale (PSS-10). For phase 2, we conducted semistructured interviews to explore user experiences; user motivation, engagement, satisfaction, and perception of the stress reduction strategies; reflections of the website’s functionality, ease of use, navigation, and design; and recommendations for improvement.

**Results:**

Google Analytics results showed 146,978 unique users from June 2020 to March 2021. Most users were from Canada (130,066, 88.5%). Between February 20, 2021, and June 4, 2021, 152 users completed the survey. Most users identified as white, female, and having at least a college degree. Based on the PSS-10 scores, most participants were experiencing moderate to high stress when they visited the website. Users rated the usability of the website as acceptable. Ten users completed in-depth interviews between May 2021 and June 2021. Positive feedback related to the content was that the website was a trustworthy source of mental health information with helpful evidence-based stress reduction strategies. Areas for improvement included the text heavy design of the website, wider dissemination/marketing, and greater accessibility of the website to meet the needs of diverse populations.

**Conclusions:**

Adding stress reduction resources to a website from a well-respected institution may be a practical method to increase awareness and access to evidence-based stress reduction resources during times of crisis, where there is severe disruption to usual health care contacts. Efforts to ensure that these resources are more widely accessed, especially by diverse populations, are needed.

## Introduction

The COVID-19 pandemic and its public health response are having a profound effect on all aspects of society, including mental health [1]. Introductions of public health measures, such as quarantine, travel restrictions, and physical closure of places of learning and businesses, were part of the response to reduce the spread of COVID-19 [2-4]. Quarantine, social isolation, and uncertainty surrounding the pandemic are known to negatively impact mental health [5]. For example, one study of individuals quarantined during the 2003 SARS pandemic in Toronto, Ontario, found that 31% of the sample developed posttraumatic stress disorder symptoms, and 29% demonstrated depressive symptom severity similar to that of a patient with clinically diagnosed depression [6].

In response to the disruption of in-person care during the current COVID-19 pandemic, health systems rapidly adopted virtual care options [7]. Where possible, care was delivered virtually by telephone or through video-conferencing platforms and web applications, which was especially true and possible with mental health conditions [7]. Not everyone needed or wanted in-person clinical services, but rather wanted accessible trustworthy psychoeducation to manage the uncertainty-related stress and anxiety associated with the disease itself and the impacts on their lives [8,9]. This led to a concern that in the absence of credible Canadian sources, most would turn to uncurated information through social media [10-12]. In Canada, the capacity of mental health and addiction services in the province of Ontario was also of concern [13]. Even before the pandemic, Ontario’s infrastructure for mental health and addiction services was overburdened and underfunded [14]. Thus, expecting this system to respond to people with mild to moderate distress was not deemed to be appropriate.

Given the growing evidence suggesting that positive outcomes can be achieved through the use of digital mental health technologies [15-20], on March 18, 2020 (the same month the World Health Organization declared COVID-19 a pandemic), the Centre for Addiction and Mental Health (CAMH), Toronto, Canada [21] launched a COVID-19 resource page on the CAMH website [22]. The website was designed to be a digital, plain language, nonstigmatizing psychoeducational resource for Canadians experiencing mild to moderate distress due to COVID-19. Stress was assumed to be universal due to the uncertainty associated with an evolving pandemic. The response would affect those with and without pre-existing mental illness to varying degrees, with the majority of the population experiencing mild to moderate distress, but in the absence of resources, this could overwhelm the health care system that was focusing on containing COVID-19. The website provides practical problem-solving tips based on cognitive behavioral therapy in both English and French. Specifically, it contains (1) a moderated discussion board, (2) written content on managing mental health during COVID-19, (3) self-assessments for anxiety using the Generalized Anxiety Disorder-7 (GAD-7) scale and perceived stress and resilience using the Perceived Stress Scale (PSS), and (4) a series of answers to frequently asked questions (FAQs) by the lay public on specific topics including intimate partner violence and grief. Those with diagnosable anxiety and depression might have more severe stress responses, which in turn hampers engagement with digital mental health interventions [23]. The lead developers (PS and RD) felt that these individuals should be directed to seek clinical services either in-person or virtually because they might need more intensive services.

The rapid development of digital strategies to minimize the negative impact of COVID-19 on public mental health has outpaced the evaluation of digital health interventions [24-26], creating a gap in our understanding of the utility and quality of the interventions [27]. In this study, we report on the reach, usability, and user perceptions of the CAMH COVID-19 website.

## Methods

### Study Design

This study used a mixed methods sequential explanatory design approach, which consisted of the following 2 distinct phases: (1) quantitative data collection and analysis and (2) qualitative semistructured interviews. To report this study, we adhered to the guidelines for Good Reporting of A Mixed Methods Study (GRAMMS) [28].

### Recruitment and Data Collection

#### Phase 1: Reach (Google Analytics) and Usability (Self-administered Survey)

In phase 1, Google Analytics data were used to determine the number of unique users, the countries and cities associated with the users, what pages they visited and in what order, and the channels (eg, Twitter and Facebook) that drove the most traffic. We collected Google Analytics data from June 2020 to March 2021.

In order to obtain additional descriptive information on who uses CAMH’s COVID-19 website tools, we placed an advertisement for this study on the website and sent a tweet letting people know. Tweets were also sent from the organizational accounts, @CAMHnews and @PSQuitSmoking, asking users/readers if they were interested in participating in the study. After consenting, participants completed a survey via Research Electronic Data Capture (REDCap), which included questions about sociodemographics; the PSS-10, a 10-item tool scored on a 5-point Likert scale ranging from 0 (never) to 4 (very often), which assesses subjective stress levels [29]; and the System Usability Scale (SUS) for assessing perceived usability of applications and services [30,31]. The SUS is a 10-item questionnaire containing response options ranging from strongly agree to strongly disagree. The decision to use the SUS tool for measuring usability was based on its validity to effectively distinguish between usable and nonusable systems [30]. The SUS can be easily administered to participants and has been shown to yield reliable results, even with small sample sizes [30]. Once the participants completed the survey, they were redirected to a new page via REDCap to provide their contact details to enter into a draw for a gift card. This was optional, and participants were able to choose not to enter the draw. Participants were also asked about their interest in participating in an interview and about consent for future communication (both optional), and contact information (email and phone number) was collected from those who were interested. Participants were informed that information collected from the survey might be used by CAMH for future research, in which case, the data were deidentified and only aggregated data were reported. We collected survey data from February 20, 2021, to June 4, 2021.

#### Phase 2: User Perceptions (Semistructured Interviews)

To explore and describe participant perceptions of the CAMH COVID-19 website, semistructured interviews were conducted from May 25, 2021, to June 29, 2021. Of the 152 participants who completed the CAMH COVID-19 website evaluation survey, 53 indicated that they were interested in being interviewed. All 53 participants were eligible to be interviewed as they visited the CAMH COVID-19 website and were over the age of 18 years. They were contacted by email/telephone, and a consent discussion was scheduled with individuals who agreed to proceed with an interview. Once participants consented and signed the informed consent using REDCap, they were booked for a 30-minute semistructured interview over the phone or a video-conferencing system. All interviews were audio recorded and transcribed verbatim. Ten interviews were conducted, and the data were assessed as adequate to permit deep case-oriented analysis that resulted in an in-depth understanding of users’ experiences with the website [32]. Several attempts were made to reach and recruit a heterogeneous sample of users for interviews. However, those who consented to participate were primarily college educated, white, and female. All participants who consented to an interview were from Ontario; thus, our sample was not representative of the overall population of Canada.

After updates were made, qualitative semistructured interviews were then used to further elaborate on the usability of the website. Usability was defined as the degree to which a program can be used easily, efficiently, and with satisfaction [33]. Specifically, the interview topic guide explored participants’ experiences with the website, including their motivation, engagement, satisfaction, or dissatisfaction, and their perceived experiences on whether the website helped to reduce their stress. It also explored participants’ reflections on the CAMH COVID-19 website’s functionality and ease of use/navigation, the overall look/design, and how the intervention could be improved.

### Timeline of Project Activities

**Figure 1:**
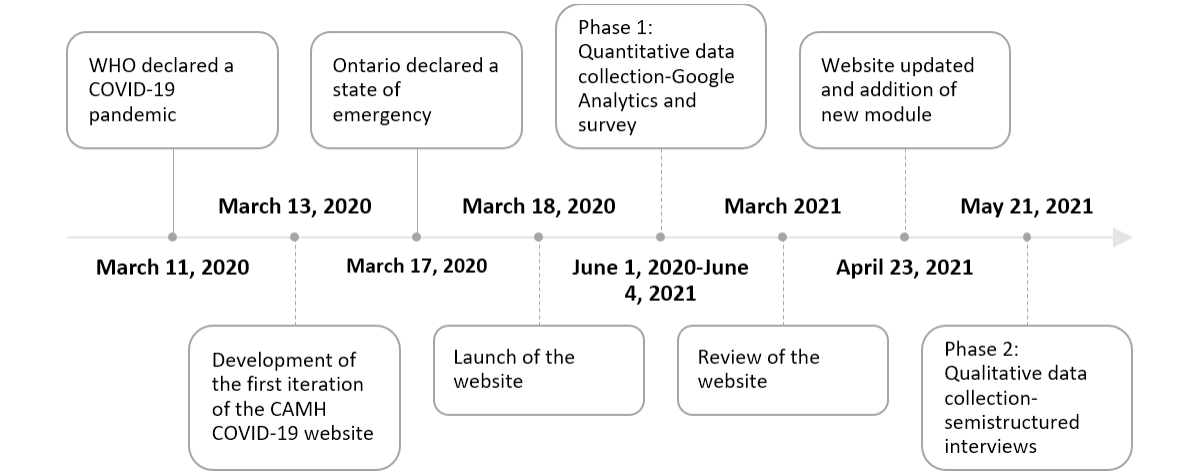
Project timeline. CAMH: Centre for Addiction and Mental Health; WHO: World Health Organization.

Figure 1 provides a timeline of project activities. The website was launched on March 18, 2020. Content was added over the year including making the website available offline through a downloadable app available on the App Store for Apple devices and Google Play Store for Android devices. Topics were added, based on the evolution of the pandemic and perceived needs to be addressed, by the planning committee (eg, parenting, grief, trauma and loss, parent support, and back to school). On April 23, 2021, based on preliminary feedback from this study and a review of the website by a trained behavioral psychologist and professional designer, the website was updated to improve navigation, eliminate duplication, and include additional content (eg, support for caregivers), based on what was going on at the time.

### Data Analysis

#### Phase 1: Google Analytics and Self-administered Survey

Google Analytics data collected from June 2020 to March 2021 were exported to Microsoft Excel, which we used to conduct descriptive statistics. We calculated means, standard deviations, and medians where applicable.

Quantitative data from the survey were analyzed using SPSS (version 25; IBM Corp). The scoring guidelines provided by the US Department of Health and Human Services were used to inform the analysis of the SUS questionnaire in the survey [30]. A SUS score between 0 and 100 was generated for each participant by converting the score for each question into a new number, summing the converted scores, and multiplying the score by 2.5. Descriptive statistics were used to report sociodemographic characteristics, usability, and engagement. The PSS-10 was analyzed by first reversing scores for 4 of the positive items on the scale and then summing across all 10 items to receive a score between 0 and 40 for each participant.

#### Phase 2: Semistructured Interviews

Qualitative data analysis for the study involved an iterative team-based process. Transcribed interviews were entered into NVivo 12 (QSR International) for qualitative data management and analysis. Two research staff read interview transcripts multiple times to achieve immersion prior to code development. The author AG crafted the initial codebook, and then, AG and AS jointly coded the first 3 transcripts to refine the codebook and defined the codes through consensus. Two other transcripts were coded independently by AG and AS, and the calculated intercoder reliability was assessed using the kappa coefficient in NVivo 12. There was 93% agreement on 96% of codes [34]. The remaining transcripts were independently coded either by AG or AS. Data were analyzed using thematic analysis [35]. A deductive approach was used to identify the coding scheme for the transcripts that allowed for the development of codes corresponding to the components of usability and stress management.

We merged both the quantitative and qualitative analysis results to provide a more comprehensive overview of participants’ perceptions of the CAMH COVID-19 website.

### Ethics

This study, including the recruitment and consent process, received ethics approval from the CAMH Quality Project Ethics Review Board (QPER #: 2020_34) [36].

## Results

### Phase 1: Google Analytics and Self-administered Survey

#### Google Analytics

From June 2020 to March 2021, there were 146,978 unique users (unduplicated/counted only once) of the website. Of the 146,978 users from all over the world, most were from Canada (130,066, 88.5%), and of these, 30,964 (23.8%) were from Toronto and 6538 (5.0%) were from Ottawa. The homepage was the most visited page of the website, which had 91,866 unique page views during the 10 months, followed by the page on “Coping with Stress and Anxiety,” which had 29,889 unique page views during the 10 months [22,37]. Organic search traffic (ie, entry through a search engine) accounted for the highest proportion of user visits to the website. From June 2020 to March 2021, 108,332 users (73.7% of all unique visitors) visited the site through organic search and 36,630 users (24.9% of all unique visitors) visited the site by typing the URL directly into their address bar.

#### Characteristics of Survey and Interview Participants

Survey responses are shown in Table 1. We have divided participant responses into 3 columns. The first column includes participants who responded between February 20, 2021, and April 23, 2021 (before the website was updated); the second includes participants who responded between April 24, 2021, and June 4, 2021 (after the website was updated); and the third includes participants we interviewed.

**Table 1:** Demographic data, Perceived Stress Scale, and System Usability Scale (SUS) scores.

Variable		Survey participants (February 20-April 23, 2021) (N=101^a^)	Survey participants (April 24-June 4, 2021) (N=51^b^)	Interview participants (May-June 2021) (N=10)
**Race/ethnicity, n (%)**				
	White	76 (75.3)	36 (70.6)	5 (50.0)
	Latin American	5 (5.0)	1 (2.0)	1 (10.0)
	Mixed (eg, African and White)	2 (2.0)	2 (3.9)	1 (10.0)
	East/South/South East Asian	6 (5.9)	6 (11.8)	2 (20.0)
	First Nations/Indigenous	0 (0.0)	1 (2.0)	0 (0.0)
	Black	3 (3.0)	1 (2.0)	0 (0.0)
	Indian–Caribbean	1 (1.0)	0 (0.0)	1 (10.0)
	Did not answer	8 (7.9)	4 (7.8)	0 (0.0)
Age (years), mean (SD)		44 (18)	45 (18)	45 (15)
**Gender, n (%)**				
	Female	86 (85.1)	39 (76.5)	10 (100.0)
	Male	10 (9.9)	7 (13.7)	0 (0.0)
	Nonbinary	0 (0.0)	4 (7.8)	0 (0.0)
	Unknown/did not know/did not answer	5 (5.0)	1 (2.0)	0 (0.0)
**Education, n (%)**				
	Graduate degree	16 (15.8)	17 (33.3)	7 (70.0)
	University degree	33 (32.7)	9 (17.6)	1 (10.0)
	College diploma	24 (23.8)	12 (23.5)	1 (10.0)
	Trade/vocational school	6 (5.9)	5 (9.8)	1 (10.0)
	High school	18 (17.8)	7 (13.7)	0 (0.0)
	Elementary school	3 (3.0)	0 (0.0)	0 (0.0)
	Did not answer	1 (1.0)	1 (2.0)	0 (0.0)
**Income (CAD $)^c^, n (%)**				
	$0-$29,999	19 (18.8)	7 (13.7)	2 (20.0)
	$30,000-$59,999	19 (18.8)	12 (23.5)	0 (0.0)
	$60,000-$89,999	24 (13.9)	5 (9.8)	2 (20.0)
	$90,000-$119,999	12 (11.9)	7 (13.7)	3 (30.0)
	$120,000-$149,999	5 (5.0)	2 (3.9)	0 (0.0)
	$150,000 or more	15 (14.9)	9 (17.6)	2 (20.0)
	Do not know/prefer not to disclose	17 (16.8)	9 (17.6)	1 (10.0)
**Marital status, n (%)**				
	Married/in a relationship	47 (46.5)	27 (54.0)	7 (70.0)
	Divorced/separated	14 (13.9)	2 (4.0)	1 (10.0)
	Single/never married	37 (36.6)	17 (34.0)	1 (10.0)
	Widowed	2 (2.0)	2 (4.0)	1 (10.0)
	Do not know/did not answer	1 (1.0)	2 (4.0)	0 (0.0)
**Level of perceived stress (Perceived Stress Scale score range), n (%)**				
	Low stress (0-13)	8 (9.0)	6 (12.5)	0 (0.0)
	Moderate stress (14-26)	44 (49.4)	25 (52.1)	6 (60.0)
	High perceived stress (27-40)	37 (41.6)	17 (35.4)	4 (40.0)
**System Usability Scale, n (%)**				
	Above average (greater than 68)	42 (48.3)	28 (58.3)	7 (70.0)
	Below average (lower than 68)	45 (51.7)	20 (41.7)	3 (30.0)

^a^For the variables age, level of perceived stress, and System Usability Scale finding, the N values were 99, 89, and 87, respectively.

^b^For the variables age, marital status, level of perceived stress, and System Usability Scale finding, the N values were 47, 50, 48, and 48, respectively.

^c^A currency exchange rate of CAD $1=US $0.77 is applicable.

#### Survey Results

In total, 152 participants completed the survey. Most participants identified as white and female, and had at least a college degree. Based on the PSS-10 scores, most participants were experiencing moderate to high stress when they visited the website.

Survey data from February 20, 2021, to April 23, 2021 (before the website was updated) indicated that participants reported a mean SUS score of 66.44 (SD 16.72; IQR 10-97.5; N=87). According to the SUS, this score was below average for a website. Specific SUS items that received a low score included the website being well-integrated (41/87, 47% agreed or strongly agreed) and the likelihood of using the website frequently (34/87, 39% agreed or strongly agreed).

After changes to the website were implemented, survey data from April 24, 2021, to June 4, 2021, indicated that the participants reported a mean SUS score of 70 (SD 17.02; IQR 22.5-100; N=48). According to the SUS, this score was above average for a website. Improved scores were reported for the website being well-integrated (31/48, 65% agreed or strongly agreed) and the likelihood of using the website frequently (26/48, 55% agreed or strongly agreed).

### Phase 2: Interview Results

#### Background on Participants Interviewed

Interview participants (n=10) were experiencing moderate to high levels of stress at the time they visited the website. Four participants had a history of mental illness or had a family member with a mental illness who received services from CAMH. Of 2 participants, whose family members received services from CAMH, one was an essential worker and the other a retired health care worker looking for mental health resources. The 2 participants with a diagnosed mental illness had recently contracted and recovered from COVID-19. Both stated that they were frequent users of the general CAMH website and visited the CAMH COVID-19 page for COVID-19–related information. Five other participants were researchers or professionals in a health-related field working with individuals who required mental health services. One participant was a stay-at-home parent with young children.

#### Functionality, Ease of Use, and Design

Most participants who were interviewed reported having visited the CAMH COVID-19 website once or twice, and having gone there to look for resources on how to deal with stress (either for themselves or for others). All participants (n=10) shared that their first impression of the website was positive, and that there were many helpful resources. Generally, participants found the website visually appealing, and liked the diversity in the images.

I liked the overall design, sort of the esthetic of the website. So, I found all of the photos that are in there to be quite diverse, in terms of the way that people look and the sort of different activities that they’re doing. They all seem very relatable….

29-year-old participant

Several participants (n=5) reported that the information was helpful, but it was not well organized. Two participants specifically mentioned feeling overwhelmed by the amount of information on the website.

Well, my immediate impressions were that it was really overwhelming. … There’s so many options…. Great…I mean it’s great to have so many options, but because of the pandemic, I think people are already overwhelmed… There’s a lot of good stuff there.

72-year-old participant

Another common theme, mentioned by 4 participants, was that the information might not be accessible to many users who might benefit from the information on the website.

My parents are refugees, like a lot of these words would be difficult for them to understand…like stigma and prejudice…I don’t know that those words mean anything to them. Maybe making things a bit more layman’s terms. Like if you want to just, kind of just speak to the masses, versus people that are in a clinical setting. Or have a background in mental health and social work.

35-year-old participant

In addition, 3 participants mentioned that there was a need to disseminate the website to those who might need it the most.

My partner, for example, he’s dealt with mental illness and homelessness for over ten years, and he didn’t know that this website existed even.

43-year-old participant

#### The Website and Stress

When asked specifically about the website as a stress reduction strategy for people in their province, all participants (n=10) responded that the website had the potential to reduce stress for individuals seeking help with stress-related issues. All participants were from Ontario. Participants noted that CAMH is a trusted authority where people can access evidence-based resources without judgement.

Well, I think it’s great that, I think it’s great that you, you know, that you’re a trusted authority. And I think it’s great…I think it’s really wonderful that all…that this website has been put up. Because, you know, I mean people need, need to get stuff from a trusted place, you know. And a place where they won’t…and where they won’t be judged, which is another huge thing is judgment, right.

72-year-old participant

I think, I really liked that you can take the tests. You know the…you know like I’m familiar with the GAD7 just because I’m a Psychologist....Like, it’s just nice to see that, you know there’s evidence-based research supported measures...

37-year-old participant

Consistent with the Google Analytics data, participants mentioned that the section they visited the most was “Stress and Anxiety.” Health professionals (n=3) indicated that the self-assessment resources found on this page would be helpful to their clients.

And, I think, when I went to the website, there were like different advice kind of little columns that you can read about how to like stay active and how to decrease your stress and your burnout and whatnot. And then there’s like different little tests you can take. And so, I thought that was…it was just really nicely laid out. Yeah. For…I mean, not for myself, like I already, you know, I’m kind of like at the point where I’m kind of feeling anxious, as given my profession, but for clients and whatnot…I think it’s helpful for them...

37-year-old participant

A participant, who was single, mentioned that she found the stress resources needed and was able to employ the recommended stress and coping strategies outlined on the website. This was in direct contrast to what parents said, as noted below.

Well, it was very good. Because it tells you, you know you need to be…keep yourself busy. You need to enjoy things like, you know hobbies…. Yes. Definitely. And I think for people who, you know like myself, who lives alone. So, I read that…I read it basically to give me information as a single person what is going on.

62-year-old participant

The need for information tailored to parents was mentioned by 2 participants as follows:

I think if you had a section for parents and that’s something I think that would be helpful for sure. When you have kids it’s like, you, you are responsible for other people than yourself. And it’s just so much harder, because there is no break …so it was something that was useful, but for me in my situation, it wasn’t quite enough, just because, you know you can’t really control your children. So, like there’s only so much that, so much that’s limited to you having control over. Right. …so the coping strategies, I think, are great, if you are a single person with no other people dependent on you.

32-year-old participant

Participants with higher levels of stress or those who described their situation as more complex noted that the stress management strategies on the website were good for “normal stress,” but that these strategies were not able to address the specific mental health needs during the COVID-19 pandemic.

I think that the stress from COVID-19, is just very…it’s very unique, in the fact that like obviously none of us have really been through a pandemic like this. But I think normal coping mechanisms may not work as well, just because the nature of COVID. …you can’t get out of the pandemic. There’s no escaping it. So, I feel like the stress management strategies were very good stress management strategies for if you had normal stress.

32-year-old participant

A suggestion by 3 participants was to have a feature that allows the user to connect to a live counsellor for more personalized support, in order to get immediate help. While access to a live counsellor was not available, the website included an online peer-to-peer “Discussion Forum” where moderators were available during business hours to provide general information and encourage the use of available resources. However, only 1 of the 10 interview participants was aware of the discussion forum feature on the website.

…the only thing that you could improve, it would if you could connect somebody with a live person. I think it should lead to someone, if somebody really needs to talk to someone, I think that they should be able to…. And I…believe me, I’ve tried a lot… I think that they should be able to connect to a live person. Because people who are desperate, need humanity.

72-year-old participant

I think one of the big things that I was kind of like getting to, was just that there wasn’t…there wasn’t an option to get help right away.

35-year-old participant

## Discussion

### Principal Findings

The goals of the study were to collect information on the reach, usability, and user perceptions of the CAMH COVID-19 website, a web page designed to be a digital, plain-language, nonstigmatizing psychoeducational resource for people experiencing mild to moderate distress due to COVID-19.

Compared with similar websites [38], the CAMH COVID-19 website [22] is a highly accessed website, with 146,978 unique individuals visiting the website in a 10-month period. The majority of the 146,978 users were from Toronto and Ottawa, which is consistent with the findings of a similar study evaluating a Canadian mental health website portal [39] and may be reflective of the increase in the percentage of Ontario residents who indicated a poor or fair mental health status during the second wave of the pandemic [40]. The most visited section was the home page [22], followed by the Coping with Stress and Anxiety [37] section, which shares different coping ideas and strategies, and includes various self-assessment tests that help users understand/determine the stress levels and anxiety they are experiencing, such as the PSS and GAD-7. Other studies have similar findings that the home page was viewed most frequently [41,42], with the second being a “self-check page, on which users could complete a depression screening tool” [41].

Users rated the usability of the website higher after changes were made on April 23, 2021. During the first period of the survey (February 20, 2021, to April 23, 2021), the average SUS score was 66.44 (SD 16.72). The SUS increased by 3.6 points after changes were made to the website (SUS score 70, SD 17.02). Both these scores correlated with an acceptable range [31,43,44] and were similar to results obtained in other evaluations of mental health websites [45].

Interview participants indicated that in the first iteration of the website, they felt overwhelmed with the volume of information and text-heavy web pages. Other studies have confirmed that text-heavy interventions can make content difficult to digest, thereby acting as a barrier to use [46], and website design related to page complexity, navigational simplicity, and comprehensibility should be considered to “reduce the cognitive effort required to effectively use eHealth applications” [47].

Demographic data indicated that the majority of website users were white women with higher education aged between 20 and 59 years. This is consistent with the findings of other studies that females and adults aged 18-64 years, with high levels of socioeconomic status, are more engaged, than males and other age groups, in using health content–related websites [39,48-54]. The website’s reach may be a reflection of recent evidence showing that women perceived a decline in their mental health in 2020 during the second wave of the pandemic and experienced worse mental health outcomes in comparison with men [1,25,40,55]. Moreover, the gender differences in the website’s reach may be attributed to men’s reluctance to access mental health care because the intervention is not sufficiently sensitive to masculine identity–related factors [56]. Given the disproportionate negative impact of COVID-19 on diverse groups [40], there is a need to reach a wider audience. Targeted strategies need to be informed by gender sensitized services [57] and are needed to provide mental health services to communities who have been disproportionately impacted by the COVID-19 pandemic, such as older adults, indigenous people, and other racial and ethnic minority groups [58]. However, research shows that these communities (older adults, indigenous peoples, and other racial and ethnic minority groups) are the least likely to seek web-based health information [25,52,54]. Proposed strategies to increase user engagement and technology acceptance of e-mental health among diverse populations include the implementation of a human-centered design approach that integrates designers and users in the development process from beginning to end [59].

Interview findings showed that most participants felt positively about the website, as it was a trustworthy source of mental health information and had helpful stress reduction features. Studies have confirmed that individuals seeking mental health resources online are concerned about the trustworthiness and credibility of e-mental health, as well as the effectiveness of self-help strategies [60-62], and as shown by another study, a lack of trust in a single online source can contribute to health information seekers’ stress [63].

Several interview participants wanted to connect to a live counsellor for more personalized support and immediate help. While moderators of the website’s “Discussion Forum” [64] offered informational support, though not live counselling and support limited to office hours, most interview participants were unaware of this feature. This may be related to the design of the website and participants’ feedback that they found the website overwhelming and text heavy. Making the discussion forum more visible might be important, especially given that research has shown that well-designed self-guided interventions can be successful without added human support and their associated costs [65-68].

The website was designed to address the needs of individuals with low to moderate levels of stress. However, the results indicated that most of the individuals visiting the website had moderate to high levels of stress. Further research is needed to understand the characteristics of individuals with low levels of stress and their low engagement with the website. Technology-enabled care coordination, which involves the use of online technology to facilitate delivery of multidisciplinary team-based care, for patients with low to moderate levels of distress has significant potential for improving population mental health [69]. Improvements to the website are needed to address the needs of individuals with high levels of stress, since studies have shown that individuals with more severe mental health symptoms are motivated to use digital mental health interventions, but symptom severity can also form barriers to engagement [23]. A guided approach, which includes the provision of content that is relevant and customizable according to users’ personal preferences and offers technical assistance or training, may be suited to address the mental health of individuals with higher levels of stress [23].

Our findings demonstrate that the CAMH COVID-19 website was useful for disseminating information about evidence-based stress reduction strategies. The website is a highly accessed website with above average usability. Users found the website to be a helpful and trustworthy source of mental health information. The recommendations suggest that the design and reach of the website need to align more closely with user needs and with greater and more equitable access to mental health care. These evaluations should be conducted with an equity-oriented approach. For example, examination of who was exposed to the website (ie, reach) allowed us to understand the need to focus on equitable reach throughout any future updates. Feedback from this evaluation will be useful for further improvements to the website, which will be beneficial to both the general public and health care professionals.

### Strengths and Limitations

An important strength of this project was the mixed methods design, as it allowed us to understand the complex problem of stress during the first waves of COVID-19 in Ontario and understand what users need as part of a website intervention. Another strength of this project is that based on direct feedback from users, we were able to make significant improvements to the usability of the website. Our study has several limitations. First, we had a convenient sample and a low number of respondents compared with the number of users in both our survey and interview. Thus, they may not have been representative samples, and it is possible that those who perceived to have gained benefits from the website were more likely to participate in the surveys and interviews. In addition, the small sample of people who agreed to be interviewed (all women) also limited the ability to stratify findings by demographics or other variables of interest. Second, participants were interviewed a number of weeks after having used the website and completing the survey. As such, for some, recalling the program details was difficult. Third, there was a lack of longitudinal follow-up data on the effects of the website. Our study was restricted to the reach, usability, and user perception of the website and did not evaluate clinical efficacy. Lastly, anxiety and depression were not addressed, as the developers felt that individuals with these diagnosable illnesses needed either in-person or virtual clinical services.

### Conclusion

Adding stress reduction resources to a website from a well-respected institution may be a practical method to increase awareness and access to evidence-based stress-reduction resources during times of crisis, where there is severe disruption to usual health care contacts. Efforts are needed to ensure that these resources are more widely accessed, especially by diverse populations. Further research is required on the health impact and role of e-mental health initiatives within the mental health services system. Given the promise and significant investment in digital mental health technologies, there is a growing need to evaluate them for low-intensity psychoeducational interventions.

## Abbreviations

CAMH: Centre for Addiction and Mental Health
GAD-7: Generalized Anxiety Disorder-7
PSS-10: Perceived Stress Scale-10
REDCap: Research Electronic Data Capture
SUS: System Usability Scale
